# Trace metals and magnetic particles in PM_2.5_: Magnetic identification and its implications

**DOI:** 10.1038/s41598-017-08628-0

**Published:** 2017-08-29

**Authors:** Jinhua Wang, Shiwei Li, Huiming Li, Xin Qian, Xiaolong Li, Xuemei Liu, Hao Lu, Cheng Wang, Yixuan Sun

**Affiliations:** 1 State Key Laboratory of Pollution Control and Resources Reuse, School of the Environment, Nanjing University, Nanjing, 210023 PR China; 2 Jiangsu Collaborative Innovation Center of Atmospheric Environment and Equipment Technology (CICAEET), Jiangsu Key Laboratory of Atmospheric Environment Monitoring and Pollution Control (AEMPC), School of Environmental Sciences and Engineering, Nanjing University of Information Science and Technology, Nanjing, 210044 China; 3 School of Earth and Environment, Anhui University of Science and Technology, Huainan, 232001 China; 4 Faculty of Chemical Engineering, Huaiyin Institute of Technology, Huaian, 223003 China

## Abstract

Magnetic measurement was combined with geochemical analysis to investigate the trace metal pollution of PM_2.5_. The study was carried out in Nanjing, China, where the average PM_2.5_ concentrations in summer and winter in 2013–2014 were 66.37 and 96.92 μg/m^3^, respectively. The dominant magnetic mineral in PM_2.5_ had a low-coercivity pseudo-single domain and consisted of magnetite and hematite. Iron-oxide magnetic particles comprised spherical as well as angular particles. Stable Pb isotopic ratio determinations showed that Pb in summer samples derived from coal emissions while the main sources of winter samples were smelting industry and coal emissions. The magnetic properties of the particles correlated strongly with trace metals derived from anthropogenic activities, such as industrial emission, coal combustion, and traffic vehicle activities, but poorly with those derived from natural sources. In the multiple linear regression analysis, Cr and Fe had higher correlation coefficients (training R > 0.7) in contrast to the low training R of As, Cd, Ni, Sr, and Ti (<0.5) determined using the PM_2.5_ concentrations and magnetic parameter values as the decision variables. Our results support the use of environmental magnetism determinations as a simple and fast method to assess trace metals in urban particulate matter.

## Introduction

Rapid industrial and social development has led to serious haze events and air pollution in China, with particles <2.5 µm in diameter (PM_2.5_) as one of the key contributors to both^1–3^. Accumulating epidemiological data suggest an association between increasing PM_2.5_ concentrations and remarkable increases in morbidity and mortality in humans^4, 5^. The sizes and concentrations of PM_2.5_ in part determine the toxic effects caused by the particles, but the chemical composition of PM_2.5_, especially their trace metal components, also play a crucial role in the severity of the associated toxic effects^6–9^. PM_2.5_ may contain high concentrations of trace metals that can enter the body after particle inhalation. The adverse health effects of trace metal exposure^10–13^ include hematological problems in the case of Pb inhalation or ingestion and damage to plasmid DNA in the case of Zn exposure^12, 14–16^. These and similar findings for other trace metals underlie the current interest in determining and assessing trace metal concentrations in PM_2.5_ and the effects that these metals may have on health.

The methods that are traditionally used to determine trace metal concentrations are atomic absorption spectrometry, inductively coupled plasma (ICP) atomic emission spectroscopy, and ICP mass spectrometry, but they are relatively complex, time-consuming, and expensive. However, the trace metal contents of various environmental matrices can also be determined using environmental magnetic methods, which are simple, rapid, nondestructive, reliable, cost-efficient, and have been used in dust^17–20^, soil^21–24^, sediment^25–27^, and plants^22, 24, 28–30^. The utility of these methods is based on the association between trace metal pollution and ferromagnetic and/or ferrimagnetic particle emissions, because Fe is abundant in natural resources^18, 24^. Nonetheless, few studies have combined trace metal analyses and environmental magnetic measurements to analyze atmospheric particles^31, 32^. The studies that have been performed have mainly focused on identifying the magnetic minerals present in the particles^31–34^. In the case of Pb, its stable isotope ratio has been successfully used to identify the sources of Pb in various environmental media, including atmospheric particles^35–37^. By contrast, systematic assessments combining analyses of the trace metal contents, magnetic properties, and Pb isotope ratios of PM_2.5_ are for the most part lacking.

The main objectives of the present study were: (1) to identify the contamination status and sources of the trace metals As, Cd, Cr, Co, Cu, Fe, Mn, Ni, Pb, Sr, Ti, V, and Zn in PM_2.5_ samples collected in a large metropolitan city in China (Nanjing), (2) to determine the magnetic characteristics of the PM_2.5_, and (3) to identify the magnetic parameters suitable for use as proxies in assessments of trace metal contamination.

## Results and Discussion

### Mass concentrations of PM_2.5_

The mean PM_2.5_ concentrations in the summer and winter sampling periods were 66.37 μg/m^3^ (range: 24.38–114.7 μg/m^3^) and 96.92 μg/m^3^ (range: 56.37–139.8 μg/m^3^), respectively. The mean PM_2.5_ concentration was significantly lower in summer than in winter (P = 0.000). PM_2.5_ is a major contributor to haze events^2, 3^ and haze was observed in Nanjing during the winter sampling period. The PM_2.5_ concentrations in most of the winter samples and a few summer samples exceeded the national ambient air quality standard (NAAQS), which is 75 μg/m^3^ (see Supplementary Fig. S1). The highest PM_2.5_ concentration (135.7 μg/m^3^) occurred on 16 January (i.e., in winter) and was almost two times higher than the NAAQS. There were no obvious differences between the PM_2.5_ concentrations in the daytime and nighttime samples (P > 0.05).

The 72-h simulated backward trajectories are shown in Supplementary Fig. S2. The air masses at all three heights arriving at Nanjing came from the southeast during the summer sampling period and had traveled over the East China Sea, Zhejiang Province, and Shanghai. The air masses at the 100 and 500 m during the winter sampling period came from the northwest and had traveled over Jiangsu, Anhui and Henan Province, whereas the air masses at 1000 m not only traveled over these provinces, but also over Xinjiang, Inner Mongolia, Ningxia, Shanxi and Shaanxi Province. Thus, PM_2.5_ pollution in Nanjing during the winter sampling period could, at least in part, be attributed to sources in Northern China, most likely the use of domestic coal heating systems^38–40^.

### Trace metal concentrations in PM_2.5_

The total trace metal concentrations in the PM_2.5_ samples are summarized in Table 1. The mean trace metal concentrations, expressed as volume-related contents in air (in ng/m^3^), generally decreased in the order Fe > Zn > Pb > Ti ≈ Mn > Cu > Cr > Ni ≈ Sr > As > Cd ≈ V > Co, consistent with the results of a study by Li *et al*.^11^. The mean As and Ni concentrations were significantly higher in the winter samples (As: 16.87 ± 3.409 ng/m^3^; Ni: 29.14 ± 7.868 ng/m^3^) than in the summer samples (9.633 ± 1.986 ng/m^3^, P = 0.000, and 8.637 ± 2.992 ng/m^3^, P = 0.000, respectively) based on PM_2.5_ air volume (unit: ng/m^3^), but for the other trace metals the concentrations did not differ between summer and winter (Table 1). There were no clear differences in the trace metal concentrations of the daytime and nighttime samples (P > 0.05).

**Table 1:** Trace metal concentrations in the PM_2.5_ samples and the trace metal enrichment factors (mean ± S.D.).

Element	All samples			Summer			Winter			Background value (μg/g)
	Content in volume (ng/m^3^)	Content in mass (μg/g)	EF	Content in volume (ng/m^3^)	Content in mass (μg/g)	EF	Content in volume (ng/m^3^)	Content in mass (μg/g)	EF	
As	13.25 ± 4.583	172.0 ± 61.81	82.80 ± 28.73	9.633 ± 1.986	162.1 ± 72.17	77.48 ± 34.70	16.89 ± 3.409	181.9 ± 49.24	88.12 ± 20.76	10
Cd	2.038 ± 1.684	25.38 ± 17.96	1034 ± 781.9	1.557 ± 0.977	25.47 ± 16.99	1018 ± 710.4	2.518 ± 2.093	25.28 ± 19.33	1050 ± 865.8	0.126
Co	0.620 ± 0.400	8.087 ± 5.706	2.986 ± 1.926	0.475 ± 0.356	7.980 ± 6.734	2.744 ± 2.039	0.764 ± 0.397	8.195 ± 4.633	3.228 ± 1.826	12.6
Cr	28.95 ± 7.265	399.3 ± 186.4	23.56 ± 7.439	27.12 ± 5.920	462.5 ± 221.9	26.62 ± 8.084	30.78 ± 8.138	336.1 ± 116.9	20.50 ± 5.341	77.8
Cu	45.35 ± 13.77	592.2 ± 193.8	125.1 ± 30.53	36.30 ± 8.501	601.9 ± 232.7	122.9 ± 32.46	54.40 ± 12.02	582.6 ± 150.7	127.2 ± 29.15	22.3
Fe	1096 ± 597.6	13553 ± 5410	2.195 ± 1.082	762.8 ± 257.0	12786 ± 5932	1.897 ± 0.709	1429 ± 658.1	14321 ± 4862	2.492 ± 1.309	30200
Mn	62.41 ± 25.72	805.3 ± 325.2	6.546 ± 2.310	49.63 ± 22.60	800.5 ± 365.9	6.253 ± 2.021	75.19 ± 22.43	810.1 ± 288.5	6.838 ± 2.585	585
Ni	18.89 ± 11.93	229.9 ± 122.5	40.43 ± 19.92	8.637 ± 2.992	144.5 ± 71.36	24.14 ± 7.959	29.14 ± 7.868	315.4 ± 101.7	56.72 ± 13.88	26.7
Pb	93.32 ± 46.48	1214 ± 580.4	228.3 ± 127.6	69.94 ± 28.13	1193 ± 649.2	219.6 ± 138.6	116.7 ± 49.92	1235 ± 518.6	237.1 ± 118.6	26.2
Sr	18.30 ± 11.39	250.1 ± 174.3	8.853 ± 5.436	18.39 ± 9.105	308.6 ± 186.1	10.68 ± 5.366	18.20 ± 13.54	191.6 ± 13.66	7.029 ± 4.986	132
Ti	69.94 ± 24.81	914.9 ± 348.7	1.000	58.42 ± 22.79	956.8 ± 419.0	1.000	81.46 ± 21.56	872.9 ± 265.1	1.000	4100
V	2.130 ± 1.278	29.82 ± 25.14	1.685 ± 1.197	2.206 ± 1.305	38.07 ± 31.10	2.056 ± 1.360	2.054 ± 1.279	21.57 ± 13.66	1.313 ± 0.896	83.4
Zn	324.5 ± 110.3	4222 ± 1546	329.3 ± 131.2	279.7 ± 91.87	4541 ± 1830	351.6 ± 158.9	369.4 ± 111.0	3902 ± 1159	307.1 ± 95.05	62.6

The As concentrations in all of the PM_2.5_ samples were above the NAAQS (GB3095-2012: 6 ng/m^3^) and the limit set by the World Health Organization (WHO: 6.6 ng/m^3^). The Cd concentrations in a few winter PM_2.5_ samples exceeded the NAAQS (GB3095-2012) and WHO limits (5 ng/m^3^). The Ni concentrations in 55% of the winter PM_2.5_ samples also exceeded the WHO limit (25 ng/m^3^), whereas those in all of the summer PM_2.5_ samples were below the WHO limit. The Mn concentrations in both the winter and the summer PM_2.5_ samples were below the WHO limits (150 ng/m^3^ for Mn). The Pb concentrations in of all the PM_2.5_ samples were below the NAAQS (GB3095-2012) and WHO [500 ng/m^3^ in total suspended particles (TSP)] limits, but the Pb concentrations in 20% of the winter PM_2.5_ samples were above the US NAAQS limit (150 ng/m^3^ in TSP). Although some of the trace metal concentrations were lower than the relevant limits for coarse PM, they could cause adverse effects when present in PM_2.5_. Whether this is indeed the case remains to be investigated in further research^13, 41^.

The mass-related trace metal concentrations (μg/g) in the PM_2.5_ samples are shown in Table 1. The As, Fe, Ni, Pb, and Zn concentrations were slightly higher in the summer than in the winter samples based on PM_2.5_ particle mass (μg/g). The concentrations of many of the trace metals (all except Co, Fe, Ti, and V) were higher than the background concentrations in topsoil in Jiangsu Province. As shown in Table 1, the enrichment factor (EF) values decreased in the order Cd > Zn > Pb > Cu > As > Ni > Cr > Sr ≈ Mn > Co ≈ Fe > V ≈ Ti, similar to the results of a study conducted in Nanjing in 2013^11^. The mean EF values for the trace metals shown in Table 1 indicated that in the PM_2.5_ Cd, Pb, Zn, and Cu were highly enriched (EF > 100) whereas As, Cr, and Ni were moderately enriched (100 < EF < 10). Cd had the highest EF, 1034 ± 781.9, but Zn, Pb, and Cu also had high mean EF values, 329.3 ± 131.2, 228.3 ± 127.6, and 125.13 ± 30.53, respectively. These results suggest that the Cd, Pb, Cu, and Zn bound to PM_2.5_ were mainly emitted from anthropogenic sources. By contrast, the mean EF values of Co, Mn, Sr, Ti, Fe and V were <10, indicating that these elements were minimally enriched and predominantly came from natural sources such as dust and soil^42^.

### Pb isotope compositions

**Figure 1:**
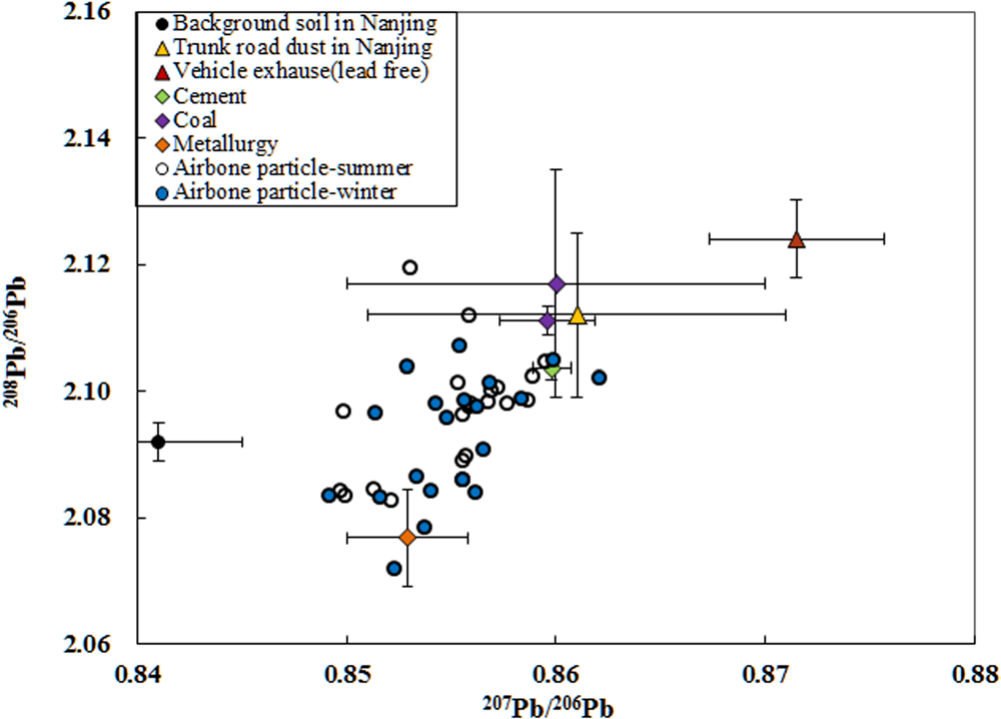
^208^Pb/^206^Pb ratios plotted against the ^207^Pb/^206^Pb ratios for the PM_2.5_ samples collected in summer and winter.

Because the ratios of anthropogenic Pb, ^208^Pb/^206^Pb and ^207^Pb/^206^Pb, are usually distinctively higher in urban environments than in natural materials^36, 43, 44^, stable Pb isotope ratios were used to identify the sources of Pb pollution in PM_2.5_ samples. The ^207^Pb/^206^Pb and ^208^Pb/^206^Pb ratios are presented in Supplementary Table S1. The scatter plots of the ^207^Pb/^206^Pb and ^208^Pb/^206^Pb ratios in the PM_2.5_ samples are shown in Fig. 1 along with the ratios of the different anthropogenic Pb sources: (lead or lead-free) vehicle exhaust, cement and metallurgy^45^, coal^37, 45^, trunk road dusts, and background soil in Nanjing^43^. The ^207^Pb/^206^Pb and ^208^Pb/^206^Pb ratios in the PM_2.5_ samples were 0.8492–0.8621 and 2.0718–2.1194, respectively. The ^207^Pb/^206^Pb and ^208^Pb/^206^Pb ratios in the winter and summer samples were not significantly different. The ratios in the PM_2.5_ samples were higher than those of background soil in Nanjing, indicating that the Pb in the PM_2.5_ samples mainly came from anthropogenic sources, consistent with the conclusions drawn from the EF value of Pb (Table 1).

The Pb isotope ratios in most of the PM_2.5_ samples were between those of coal combustion ash and metallurgical industry dust (Fig. 1), implicating these sources in the Pb pollution in Nanjing, as suggested in previous studies^41, 46^. According to the Nanjing Statistical Yearbook 2012, 80.5% of the energy consumed in Nanjing is produced by burning coal^44^. The Pb isotope ratios were similar to those in metallurgical industry dust in only a few of the summer PM_2.5_ samples; thus smelting industry activities were not the main sources of Pb in the summer sampling period. This can be explained by the fact that many smelters had been shut down before the Youth Olympic Games were held, in 2014. The Pb isotope ratios for several PM_2.5_ samples were similar to those of cement dust, consistent with the location of the sampling site in a new urban development zone in which construction work was taking place. The Pb isotope ratios were distinctly lower in the PM_2.5_ samples than in exhaust emissions from vehicles using unleaded gasoline. However, Pb is used as a tracer of non-exhaust traffic emissions^43^, and the stable Pb isotope ratios for dust emitted on trunk roads overlapped with those of some of the PM_2.5_ samples, indicating that traffic contributes to Pb pollution in Nanjing.

### Magnetic properties of PM_2.5_

The means, standard deviations, and ranges of the magnetic parameters for the summer and winter samples are shown in Table 2. The low frequency susceptibility (χ_LF_) and saturation isothermal remanent magnetization (SIRM) generally reflect the concentrations of magnetic minerals, especially ferromagnetic minerals (e.g., magnetite)^47^. Unlike SIRM, χ_LF_ is influenced by paramagnetic and diamagnetic minerals^47^, attributable to the seasonally different sources of PM_2.5_ and seasonally different magnetic mineral species found in PM_2.5_. The mean values of χ_LF_, ‘hard’ isothermal remanent magnetization (HIRM), and anhysteretic remanent magnetization susceptibility (χ_ARM_) were slightly higher in the winter than in the summer samples. However, the differences between the summer and winter χ_LF_, HIRM, χ_ARM_, and SIRM values were not significant.

**Table 2:** Magnetic parameters for the PM_2.5_ samples.

Magnetic properties	All samples				Summer				Winter			
	Mean	S.D.	Min	Max	Mean	S.D.	Min	Max	Mean	S.D.	Min	Max
χ_LF_ (×10^−8^m^3^/kg)	626.7	425.3	130.8	1900	603.7	224.7	320.9	1047	649.6	565.4	130.8	1900
χ_ARM_ (×10^−8^m^3^/kg)	1077	716.5	244.4	3209	976.3	337.8	474.7	1587	1179	958.2	244.4	3209
SIRM (×10^−5^Am^2^/kg)	51983	34153	10073	146957	54182	21395	24008	105970	49784	43890	10073	146957
HIRM (×10^−5^Am^2^/kg)	1603	1307	168.8	5965	1499	738.5	704.0	3687	1709	1715	168.8	5965
χ_ARM_/χ_LF_	1.760	0.307	1.250	2.477	1.644	0.275	1.250	2.420	1.876	0.298	1.297	2.477
χ_ARM_/SIRM (×10^−5^mA^−1^)	21.91	6.383	14.25	44.16	18.60	4.222	14.25	28.55	25.23	6.535	14.28	44.16
SIRM/χ_LF_ (×10^3^Am^−1^)	839.5	160.4	338.8	1215	900.2	116.7	646.5	1043	778.7	177.3	338.7	1215
S-ratio	97.11	0.685	95.43	98.32	97.27	0.415	96.33	98.07	96.94	0.857	95.43	98.32
L-ratio	0.221	0.038	0.146	0.304	0.216	0.027	0.171	0.255	0.227	0.047	0.146	0.304

**Figure 2:**
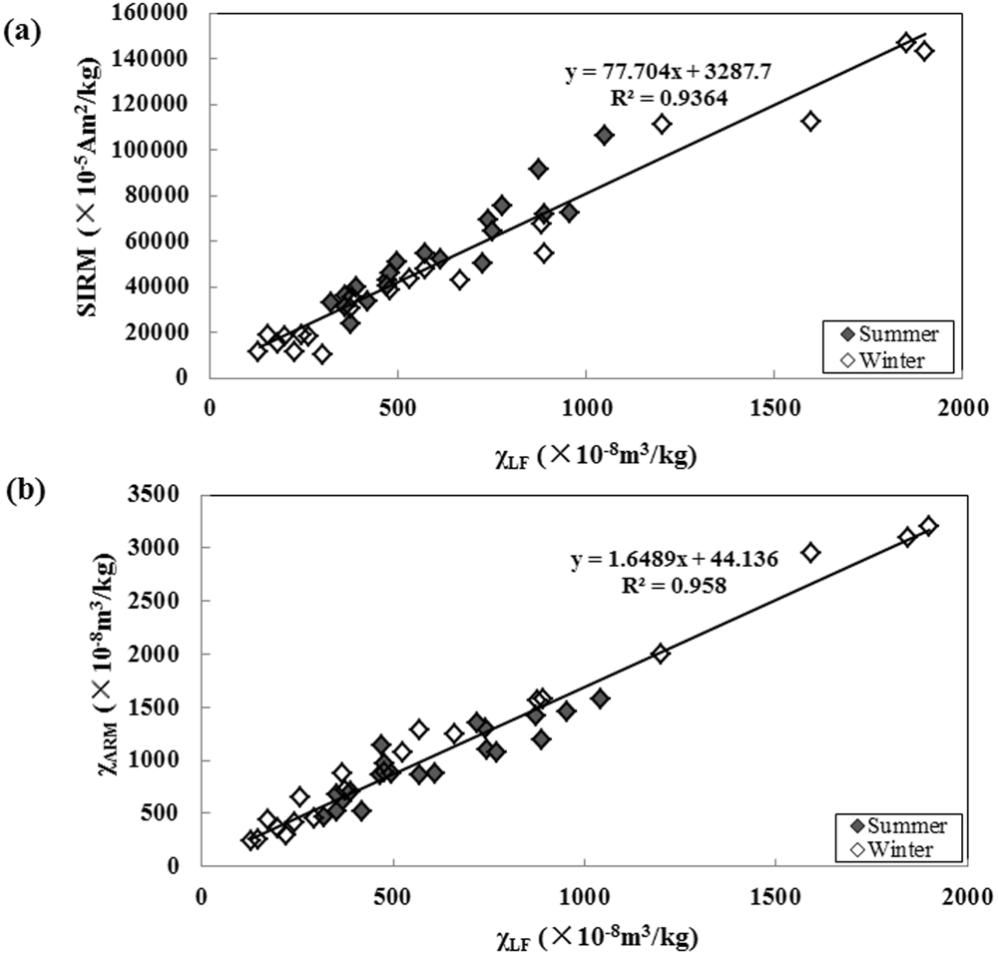
χ_LF_ plotted against (**a**) the saturation isothermal remanent magnetization value and (**b**) χ_ARM_ for the PM_2.5_ samples collected in Nanjing.

**Figure 3:**
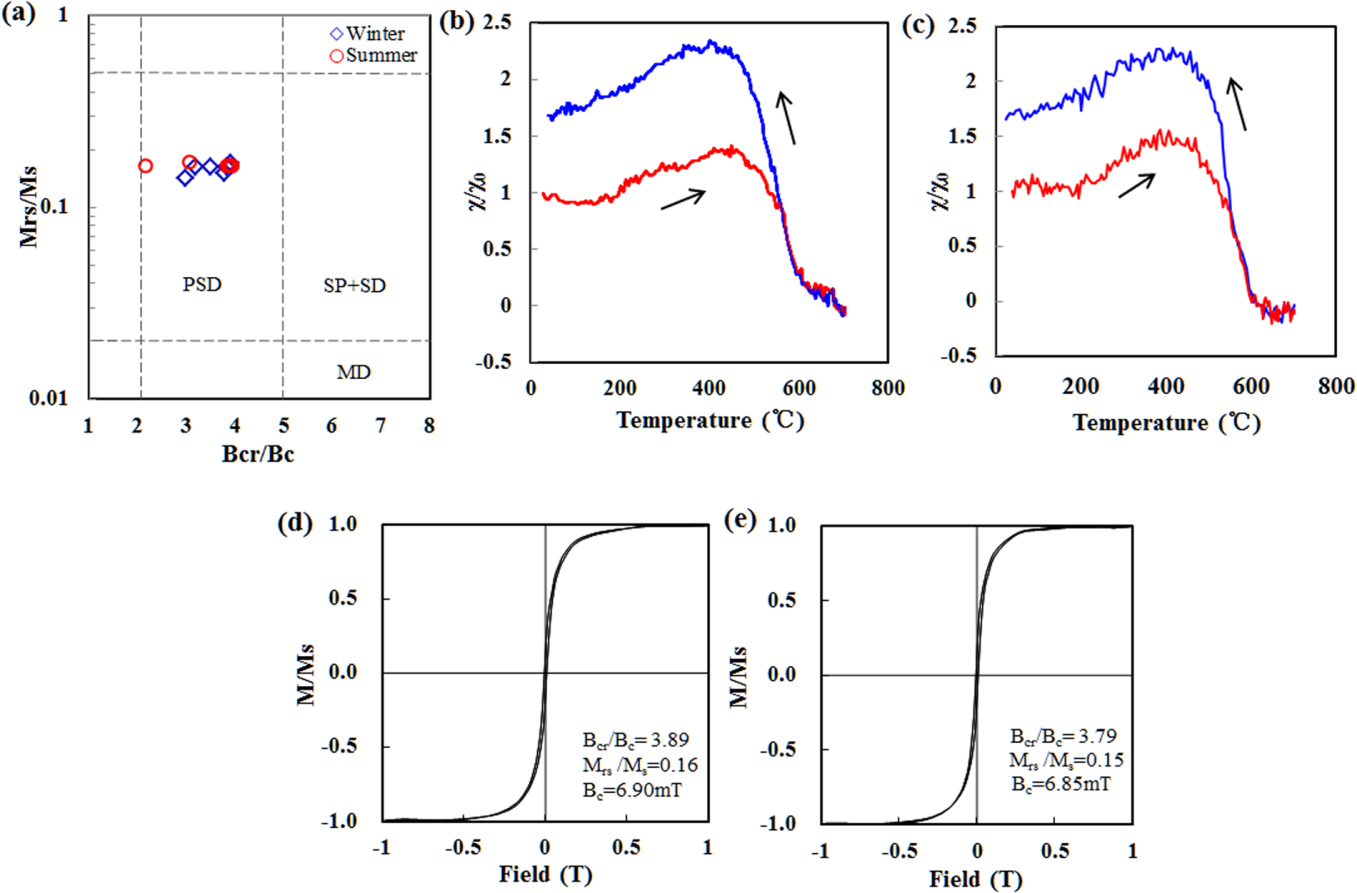
(**a**) Day plot for 12 typical samples. The blue squares and red circles are data points for the winter (six samples) and summer (six samples), respectively. (**b**) Temperature dependence of the magnetic susceptibility (χ–T) curves for selected summer PM_2.5_ samples. (**c**) Temperature dependence of the magnetic susceptibility (χ–T) curves for selected winter PM_2.5_ samples. The heating branches are shown in red and the cooling branches in blue. Each curve was normalized to the corresponding magnetic susceptibility at room temperature (χ_0_). (**d**,**e**) Hysteresis loop for (**d**) the summer and (**e**) the winter PM_2.5_ samples.

The SIRM and χ_LF_ values of all PM_2.5_ samples correlated linearly (R^2^ = 0.9364), as shown in Fig. 2a, and indicated that the dominant magnetic minerals in the PM_2.5_ samples were ferrimagnetic^48^. The χ_ARM_ may constrain the sizes of ferrimagnetic grains because of its sensitivity to single-domain and pseudo-single-domain magnetic particles^49^. The significant correlation between the χ_ARM_ and χ_LF_ values (R^2^ = 0.958, Fig. 2b) suggested that the significant increases in the χ_LF_ values were closely related to coarse ferrimagnetic phases. This was confirmed by the Day plot (Fig. 3a), which showed that either a pseudo-single-domain mineral or a mixture of single-domain and multiple-domain magnetite dominated the PM_2.5_ samples collected in both seasons. The results in a study of PM_2.5_ in Beijing were similar^34^.

The temperature-dependent susceptibility (χ−T) cycles and hysteresis loops for representative winter and summer samples are shown in Fig. 3. The χ−T curves were used to identify the Curie temperature for typical PM_2.5_ samples in summer and winter (Fig. 3b,c). A Curie temperature of ~580 °C was determined for the PM_2.5_ samples based on χ−T measurements. This value was compatible with the results expected for magnetite. The χ value decreased slightly between 600 and 700 °C, revealing the presence of hematite. The χ value increased before a temperature of 300 °C was reached, possibly because fine-grained ferrimagnetic particles gradually became unblocked. A decrease in the χ value after 300 °C is generally interpreted as reflecting the conversion of maghemite to hematite^50^. The hysteresis loops for the PM_2.5_ sample in a field of ~300 mT were thin, closed, and approached magnetic saturation (Fig. 3d,e), indicative of the presence of magnetic minerals in the samples. The S-ratios were high (96.33–98.07% in summer and 95.43–98.32% in winter), which confirmed the presence of soft, low-coercivity magnetite-type ferromagnetic minerals in the PM_2.5_ samples^47^. The L-ratio values were low and relatively stable (17.1–25.5% in summer and 14.6–30.4% in winter), which also indicated that soft ferromagnetic minerals dominated the magnetic remanence (Table 2).

**Figure 4:**
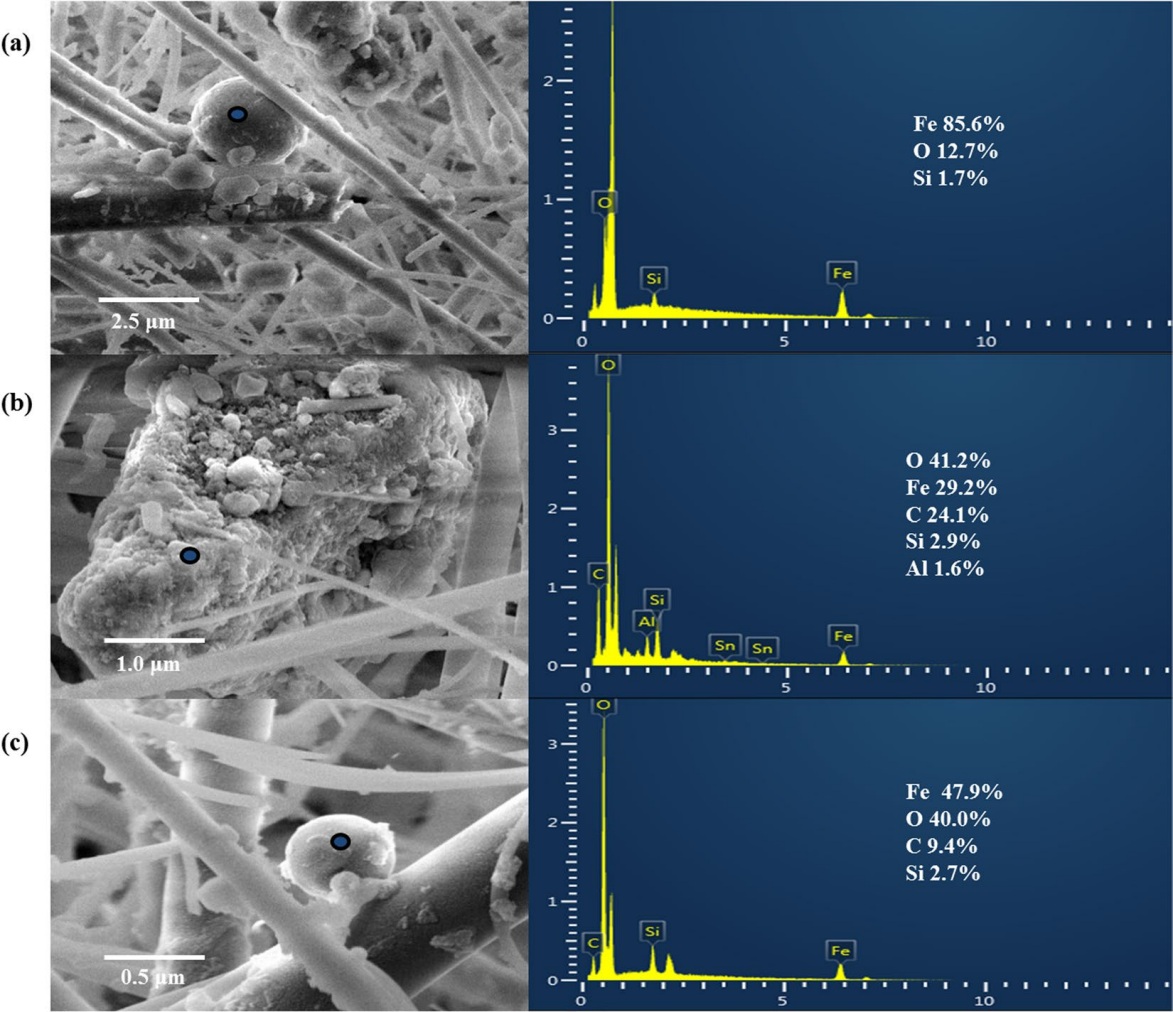
Scanning electron microscopy images and energy dispersive X-ray spectroscopy results for representative PM_2.5_ samples.

The optical characteristics of representative PM_2.5_ samples were determined using a FEI Quanta 250 FEG scanning electron microscope (Thermo Fisher Scientific, Waltham, MA, USA) with an AZtec X-Max 80 energy dispersive X-ray spectroscopy (EDX) system (Oxford Instruments, Abingdon, UK). Most of the magnetic iron oxide particles were either spherical (Fig. 4a,c) or angular (Fig. 4b). EDX analysis revealed that the Fe content of these magnetic particles was 29.2–85.6%. The spherical particles were probably produced during the combustion of fossil fuels, whereas the angular iron oxide particles containing Al, C, and Si probably came from automobile brakes or soil dust^17, 34^.

### Relationship between magnetic properties and trace metal concentrations

The mass concentrations of trace metals can reflect the variations in particle sources and allow particle toxicities to be assessed accurately. In efforts to control atmospheric pollution, it is essential to obtain trace metal mass concentration data. We therefore used trace metal mass concentrations (in μg/g) to assess the relationships between the magnetic properties and trace metal contents of the samples. As shown in Supplementary Table S2, the As, Cr, Cu, Fe, Mn, Pb, and Zn concentrations positively and significantly correlated with each other, indicating their common sources. The Cd concentration, which was extremely enriched (as mentioned above and shown in Table 1), correlated significantly with the Fe, Pb, and Zn concentrations. The Co, Sr, Ti, and V positively correlated with each other, indicating their similar natural sources^42^. The PM_2.5_ concentration correlated negatively with the Cr, Cu, Sr, Ti, V, and Zn concentrations.

The Cd and Fe concentrations positively and significantly correlated with the χ_LF_, SIRM, HIRM, and χ_ARM_ values. The Mn concentration correlated with the χ_LF_ value, and the Pb concentration significantly with the χ_LF_, HIRM, and SIRM values. The Ni concentration correlated positively and significantly with the χ_ARM_/SIRM ratio but negatively with the SIRM/χ_LF_ ratio. The correlation coefficients were higher for the relationships between the SIRM values and the Fe and Pb concentrations than for the relationships between the χ_LF_ value and the concentrations of these same metals. This could be explained by the influence on the SIRM of the ferrimagnetic and incomplete anti-ferromagnetic mineral concentrations, with the contributions of the latter under-represented and the contributions of pedogenic superparamagnetic particles not taken into account^26, 51^. This finding agreed with the results of previous studies and suggested that, as an indicator of the presence of trace metals, the SIRM value is more effective than the χ_LF_ value^17, 52, 53^. The PM_2.5_ concentration correlated positively with the χ_ARM_/χ_LF_ ratio and the L-ratio but negatively with the S-ratio.

The interrelationships of the PM_2.5_ concentrations, trace metal contents, and magnetic parameters were further examined in a multiple regression analysis. As shown in Table 3, the training R values of Cr and Fe were > 0.7, in contrast to the low correlation coefficients of As, Cd, Ni, Sr, and Ti (R < 0.5). The test R values of Fe, Pb, Sr, Mn, and Cd were relatively higher (R > 0.5). The pollution levels for most of the trace metals (except Co, the concentration of which did not correlate significantly with either the PM_2.5_ concentration or the magnetic parameters) could be linearly determined from the PM_2.5_ concentration and the magnetic parameters.

**Table 3:** Multiple linear regression results of the trace metal concentrations in the PM_2.5_ samples.

Regression equation	Training		Test
	R	p value	R
As = 265.5 − 1.166 PM_2.5_	0.463	0.010	0.191
Cd = 13.91 + 0.018 χ_LF_	0.429	0.018	0.809
Cr = 842.8 − 5.534 PM_2.5_	0.738	0.000	0.452
Cu = 892.9 − 4.034 PM_2.5_	0.570	0.001	0.149
Fe = 7461 + 0.119 SIRM	0.705	0.000	0.814
Mn = 514.7 + 0.540 χ_LF_	0.554	0.002	0.533
Ni = 64.43 + 7.094 χ_ARM_/SIRM	0.449	0.013	0.381
Pb = 908.5 + 0.021 SIRM − 0.670 χ_ARM_	0.560	0.006	0.703
Sr = 426.1 − 2.117 PM_2.5_	0.419	0.021	0.666
Ti = 1445 − 6.937 PM_2.5_	0.480	0.007	0.273
V = 20.85 − 0.434 PM_2.5_ + 23.73 χ_ARM_/χ_LF_	0.614	0.002	0.343
Zn = 6327 − 27.23 PM_2.5_	0.520	0.003	0.164

The relationships between the PM_2.5_ concentrations, trace metal concentrations, and magnetic parameters were analyzed in a principal components analysis. The results are shown in Table 4. Four principal components explained 72.61% of the variance. The first component explained 30.61% of the total variance. The relatively high first component values of the As, Cr, Cu, Fe, Mn, Pb, and Zn concentrations and the SIRM value (0.577) suggested similar anthropogenic activities, such as coal combustion, smelting, and traffic emissions, as the sources of both the trace metals and the SIRM^18, 26, 54^. Factor 2 was dominated by the PM_2.5_ concentration, the χ_LF_, SIRM, HIRM, and χ_ARM_ values, the L-ratio, and the S-ratio and explained 22.97% of the total variance. Only the S-ratio had a negative factor 2 value. Factor 3 explained 10.94% of the total variance and was dominated by the Ni concentration and the χ_ARM_/SIRM and SIRM/χ_LF_ ratios. The Ni concentration and the χ_ARM_/SIRM ratio had positive factor 3 values, but the SIRM/χ_LF_ ratio had a negative factor 3 value. The χ_ARM_/SIRM and SIRM/χ_LF_ ratios are mainly related to the magnetic mineralogy and grain size. Factor 4 explained 8.09% of the total variance and was dominated by the L-ratio.

**Table 4:** Principal component analysis results for the PM_2.5_, trace metal concentrations, and magnetic parameters (loadings > 0.6 are shown in bold).

Factor	Component			
	1	2	3	4
PM_2.5_	−0.441	0.470	0.418	0.263
As	**0.678**	−0.324	0.208	0.269
Cd	0.517	0.442	−0.063	−0.186
Co	0.414	−0.482	0.147	−0.078
Cr	**0.735**	−0.521	−0.069	0.132
Cu	**0.763**	−0.436	0.136	0.215
Mn	**0.696**	−0.100	0.400	−0.055
Ni	0.213	−0.307	**0.714**	0.417
Pb	**0.757**	0.071	0.052	0.080
Ti	0.561	−0.393	−0.212	−0.164
Sr	**0.682**	−0.335	−0.035	0.338
V	0.596	−0.365	−0.088	−0.187
Zn	**0.798**	−0.165	−0.069	−0.057
Fe	**0.808**	0.373	0.130	0.041
χ_LF_	0.530	**0.762**	0.104	−0.302
SIRM	0.577	**0.758**	−0.064	−0.201
HIRM	0.506	**0.829**	0.020	−0.016
χ_ARM_	0.422	**0.808**	0.183	−0.278
χ_ARM_/χ_LF_	−0.383	0.048	0.393	0.231
χ_ARM_/SIRM	−0.302	−0.125	**0.851**	−0.229
SIRM/χ_LF_	0.083	0.091	−**0.755**	0.481
S-ratio	−0.064	−**0.722**	−0.005	−**0.601**
L-ratio	0.032	0.592	0.031	0.586
Initial Eigenvalues	7.040	5.284	2.515	1.860
% Variance	30.61	22.97	10.94	8.09
Cumulative %	30.61	53.58	64.52	72.61

Extraction method: principal component analysis.

^a^4 components extracted.

### Environmental implications

The use of magnetic measurements to determine trace metal contamination assumes a relationship between the magnetic parameters and the trace metal concentrations. Specifically, the two most important factors are the relationships between the magnetic parameters and the Fe concentration and between the Fe concentration and the concentrations of the other trace metals^17^. In our study, the Fe concentration correlated strongly with both the concentrations of most of the anthropogenic trace metals (e.g., Cd, Mn, and Pb) and the magnetic parameters, that is, the χ_LF_, SIRM, HIRM, and χ_ARM_ values, indicating that the magnetic parameters could be used to assess trace metal pollution in PM_2.5_. However, Ni, Co, and V have multiple sources such that their concentrations correlated poorly with the magnetic parameters. According to these results, the link between magnetic properties and trace metal concentrations mainly depends on the sources of the trace metals and the magnetic minerals. In previous studies, the relationships between magnetic properties and trace metal concentrations were typically investigated using the χ_LF_ and SIRM values^26^. Our study showed that the χ_ARM_/SIRM and SIRM/χ_LF_ ratios correlated strongly with the Ni and therefore that comprehensive magnetic measurements should be performed before trace metal contamination is assessed.

### Conclusions

PM_2.5_ samples were collected in Nanjing in summer and winter. The PM_2.5_ concentrations in almost all of the winter samples and a few of the summer samples exceeded the NAAQS. The mean As and Ni concentrations were significantly higher in the winter than in the summer samples (P < 0.05). The As concentrations in all of the PM_2.5_ samples were higher than the limits set by the NAAQS and WHO. The Cd, Ni, and Pb concentrations in a few of the winter PM_2.5_ samples exceeded the NAAQS and WHO limits. These findings led us to conclude that some trace metals (As, Cd, Cr, Cu, Ni, Pb, and Zn) in our PM_2.5_ samples came from anthropogenic sources but that Co, Sr, and V (EF < 10) predominantly came from natural sources. The stable Pb isotope ratios pointed to coal combustion emissions as the main source of Pb in the PM_2.5_ from the summer samples, and to smelting and coal combustion emissions as the main sources of Pb in the winter PM_2.5_ samples.

Detailed analyses of the magnetic properties of the PM_2.5_ revealed that the dominant magnetic minerals were pseudo-single-domain magnetite in soft, low-coercivity particles. The magnetic minerals in the PM_2.5_ mainly consisted of magnetite and hematite. Scanning electron microscopy-EDX analysis showed that most of the iron oxide magnetic particles were spherical and angular particles with a high Fe content. The magnetic properties (χ_LF_, SIRM, HIRM, and χ_ARM_ values) correlated strongly with the concentrations of trace metals derived from anthropogenic activities (Cd, Fe, Mn, and Pb) but poorly with the concentrations of trace metals derived from natural processes. Multiple linear regression models for the Cr and Fe had high correlation coefficients (training R > 0.7), and those for the As, Cd, Ni, Sr, and Ti low correlation coefficients (training R < 0.5), using the PM_2.5_ concentrations and magnetic parameters as decision variables. Taken together, these results demonstrate the potential of magnetic measurements as efficient proxies to assess trace metal pollution in urban PM_2.5_.

## Methods

### Sample collection

Nanjing City (118° 46′ E, 32° 03′ N) is the capital of Jiangsu Province and the second largest city in the Yangtze River delta region. It covers approximately 6587 km^2^ and has a population of 8.2 million. Nanjing has a northern subtropical monsoon climate, and the wind predominantly comes from the southeast in summer and the northwest in winter. There are five dominant industries in Nanjing, the automobile, electric power production, electronics, petrochemical, and steel industries. In addition, Nanjing is the main transport hub in eastern China. PM_2.5_ samples were collected in Aoti, in the center of the new urban districts of Nanjing (see Supplementary Fig. S3).

One sample was collected during the day (from 08:00 to 18:00) and another during the night (from 19:00 to 07:00) on each consecutive clear day between 8 and 23 July 2013 (i.e., in summer) and between 7 and 17 January 2014 (i.e., in winter). The samples were collected using a high-volume air sampler (TE6070, Tisch Environmental, Cleves, OH, USA) and at a flow rate of 1.13 m^3^/min. Each sample was collected on a Whatman quartz microfiber filter measuring 203 mm × 254 mm (GE Healthcare Bio-Sciences, Pittsburgh, PA, USA). No samples were collected when the weather was wet and windy (which can occur in both summer and winter in Nanjing) to ensure that the samples were representative. The sampler was placed on the rooftop of an open three-story building (Fig. 1). A total of 42 PM_2.5_ samples were collected. Each filter was conditioned for 48 h in a desiccator at 25 °C and 40% relative humidity before and after sampling, and then weighed using a microbalance (Mettler-Toledo, Greifensee, Switzerland).

It should be noted that pollution controls implemented to improve air quality for the 2014 Youth Olympic Games in Nanjing (16–28 August 2014) decreased the PM_2.5_ concentrations in the atmosphere. Before the Youth Olympic Games started, the Nanjing local government implemented several pollution control measures, including closing approximately 2630 construction sites, decreasing the outputs of heavy industrial factories (such as iron and steel plants and petrochemical plants) by 20%, and banning high emission vehicles (such as trucks and heavy-duty vehicles). Industrial plants emitting large amounts of pollutants were also closed in 22 nearby cities in order to cooperate with the Nanjing local government^41^.

### Magnetic measurements

The blank filters and corresponding PM_2.5_ samples were weighed, after which each filter was placed in a nonmagnetic plastic cylinder with a volume of 11.15 cm^3^. The magnetic susceptibility at a low frequency (976 Hz) (χ_LF_) of each filter was measured and then mass normalized to obtain the χ_LF_ value using an MFK1-FA Kappabridge system (AGICO, Brno, Czech Republic) at room temperature. Anhysteretic remanent magnetization (ARM) was applied using a DTECH 2000 AF demagnetizer (ASC Scientific, Carlsbad, CA, USA), a peak alternating field of 100 mT, and a direct current bias field of 0.04 mT. The measurements were expressed as the ARM susceptibility (χ_ARM_), obtained by dividing the remanence by the steady field value. Isothermal remanent magnetization (IRM) experiments were performed using an MMPM10 pulse magnetizer (Magnetic Measurements, Aughton, UK). IRM was measured using a JR-6 dual speed spinner magnetometer (AGICO). The IRM measured in a field of 1 T is called the saturation isothermal remanent magnetization (SIRM)^33^. The IRM measured in a reverse field was expressed as a percentage of the reverse saturation of the SIRM, and the S-ratio and L-ratio were calculated using the equations S-ratio = −IRM_−300 mT_/SIRM and L-ratio = (SIRM + IRM_−300 mT_)/(SIRM + IRM_−100 mT_). The ‘hard’ IRM (HIRM) was calculated using the equation: Mass-specific HIRM = [(SIRM + IRM_−300mT_)/2]/mass^19, 55^. Hyteresis loops and magnetic hysteresis parameters, including the saturation magnetization (M_s_), saturation remanent magnetization (M_rs_), coercivity (B_c_), and remanent coercivity (B_cr_), of representative samples were measured at room temperature using an MMVFTB multi-purpose magnetic measurement system. High-temperature thermomagnetic analyses were performed using an MFK1-FA Kappabridge system equipped with a CS-3 high-temperature furnace (AGICO). Each sample was heated from room temperature to 700 °C and then cooled in an argon atmosphere to room temperature. The magnetic measurements were performed at the State Key Laboratory of Estuarine and Coastal Research, East China Normal University, Shanghai, China.

### Total trace metal analysis

Each PM_2.5_ sample was digested in ultra-pure concentrated HNO_3_ and 30% H_2_O_2_ to allow the total As, Cd, Co, Cr, Cu, Fe, Mn Ni, Pb, Sr, Ti, V, and Zn concentrations to be determined. The method was a slight modification of US Environmental Protection Agency method 3050B^41^. Briefly, about 1/16 of a quartz filter was cut into even smaller pieces (<2 mm^2^) using a clean ceramic knife. The pieces were then immersed in 10 mL of ultra-pure concentrated HNO_3_ (final acid to filter volume ratio of 1:1), ensuring that the filter pieces containing the PM_2.5_ were fully in contact with the digestion solution. The mixture was digested at 105 °C for 5 h, after which a further 10 mL of concentrated HNO_3_ was added and the mixture was digested again. The digest was allowed to evaporate almost to dryness and allowed to cool. After the addition of 2 mL of 30% H_2_O_2_, the solution was evaporated to <1 mL. The extract was then diluted to 50 mL with Milli-Q water. The concentrations of the elements of interest in the extracts were determined using an ICP-mass spectrometer (Perkin Elmer, Waltham, MA, USA). Quality assurance and quality control procedures included analyzing blank quartz microfiber filters, analytical duplicates, and standard reference material SRM 1648a (urban particulate matter) (US National Institute of Standards and Technology, Gaithersburg, MD, USA). The recovery rates were within ±10%, as recommended by the US Environmental Protection Agency^56^. The concentration of each trace element in each PM_2.5_ sample was corrected by subtracting the mean concentration of the element in the blanks.

### Stable isotope ratios in PM_2.5_

The stable Pb isotope ratios ^206^Pb/^204^Pb, ^207^Pb/^206^Pb, and ^208^Pb/^206^Pb in the total Pb extracted from each PM_2.5_ sample were determined using ICP-mass spectrometry. The trace metal extract of each PM_2.5_ sample was diluted with 0.1 M high-purity HNO_3_ to a Pb concentration of 15 μg/L. The instrument parameters were 190 sweeps/reading, 1 reading/replicate, a dwell time of 40 ms for ^204^Pb, and a dwell time of 25 ms for ^206^Pb, ^207^Pb, and ^208^Pb. Each PM_2.5_ sample was measured 10 times, and the mean Pb isotope ratio and the relative standard deviation were then calculated. The isotope intensities of the blank quartz microfiber filter samples were subtracted from the isotope intensities of each PM_2.5_ sample. The common Pb isotope standard reference material SRM 981 (US National Institute of Standards and Technology) was analyzed as a quality control procedure. The SRM 981 extract was diluted to a Pb concentration of 15 μg/L and run after every five samples to provide ratio correction factors to allow mass bias correction factors for the isotope ratios to be determined. The relative standard deviation for 10 replicate PM_2.5_ samples was generally <0.5% for both the ^207^Pb/^206^Pb and the ^208^Pb/^206^Pb ratios.

### Back trajectory analysis

The Hybrid Single-Particle Lagrangian Integrated Trajectory (HYSPLIT-4.8) model, developed by the US National Oceanic and Air Administration Air Resources Laboratory, was used to calculate 72-h air mass back trajectories using 3-hourly archived meteorological data from the US National Centers for Environmental Prediction Global Data Assimilation System^57^. Back trajectories were calculated for both sampling periods at heights of 100, 500, and 1000 m above ground level.

### Statistical analyses

The enrichment factor (EF) was calculated to evaluate the degrees to which the PM_2.5_ samples were polluted with trace metals^58, 59^. The EF was calculated using Eq. (1) in the Supplementary Information. One-way analysis of variance, Pearson’s correlation coefficient analysis, and principal components analysis to evaluate the data and multiple linear regressions were performed using SPSS version 16.0 for Windows. Multiple linear regression analysis was used to develop a linear model according to Eq. (2) (see the Supplementary Information). The data were partitioned at random into two sets: 75% for the training set and 25% for the test set.

## Electronic supplementary material

Supplementary Information
