# Supplementary material for: Trace metals and magnetic particles in PM2.5: Magnetic identification and its implications
Source: Sci Rep. 2017 Aug 29;7:9865. doi: 10.1038/s41598-017-08628-0 (PMC5574900; doi:10.1038/s41598-017-08628-0)
Supplement: Supplementary file 1 — Supplementary Information [file 41598_2017_8628_MOESM1_ESM.doc]

**Supplementary Information**

**Trace metals and magnetic particles in PM_2.5_: Magnetic identification and its implications**

Jinhua Wang^a^, Shiwei Li^a^, Huiming Li^a,b^*, Xin Qian^a,b^*, Xiaolong Li^a,c^, Xuemei Liu^a,d^, Hao Lu^a^, Cheng Wang^a^, Yixuan Sun^a^

^a^ *State Key Laboratory of Pollution Control and Resources Reuse, School of the Environment, Nanjing University, Nanjing 210023, PR China.*

^b^ *Jiangsu Collaborative Innovation Center of Atmospheric Environment and Equipment Technology (CICAEET), Jiangsu Key Laboratory of Atmospheric Environment Monitoring and Pollution Control (AEMPC), School of Environmental Sciences and Engineering, Nanjing University of Information Science and Technology, Nanjing 210044, China.*

^c^ *School of Earth and Environment, Anhui University of Science and Technology, Huainan 232001, China.*

^d^ *Faculty of Chemical Engineering, Huaiyin Institute of Technology, Huaian 223003, China.*

^*^Corresponding authors: Huiming Li and Xin Qian. Address: State Key Laboratory of Pollution Control and Resources Reuse, School of the Environment, Xianlin Campus, Nanjing University, Jiangsu Nanjing 210023, P. R. China. Tel: +86-25-89680527, +86-25-83686522. *E-mail addresses*: [valen222@126.com](mailto:valen222@126.com), [xqian@nju.edu.cn](mailto:xqian@nju.edu.cn).


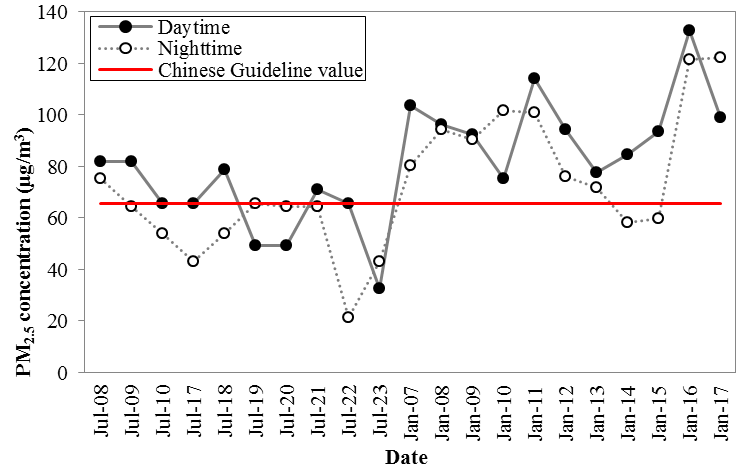


**Supplementary Figure S1.** PM_2.5_ concentrations during the daytime (08:00–18:00) and nighttime (19:00–07:00). The red line shows the 75 μg/m^3^ 24-h standard established in the new Chinese National Ambient Air Quality Standard (GB3095-2012).


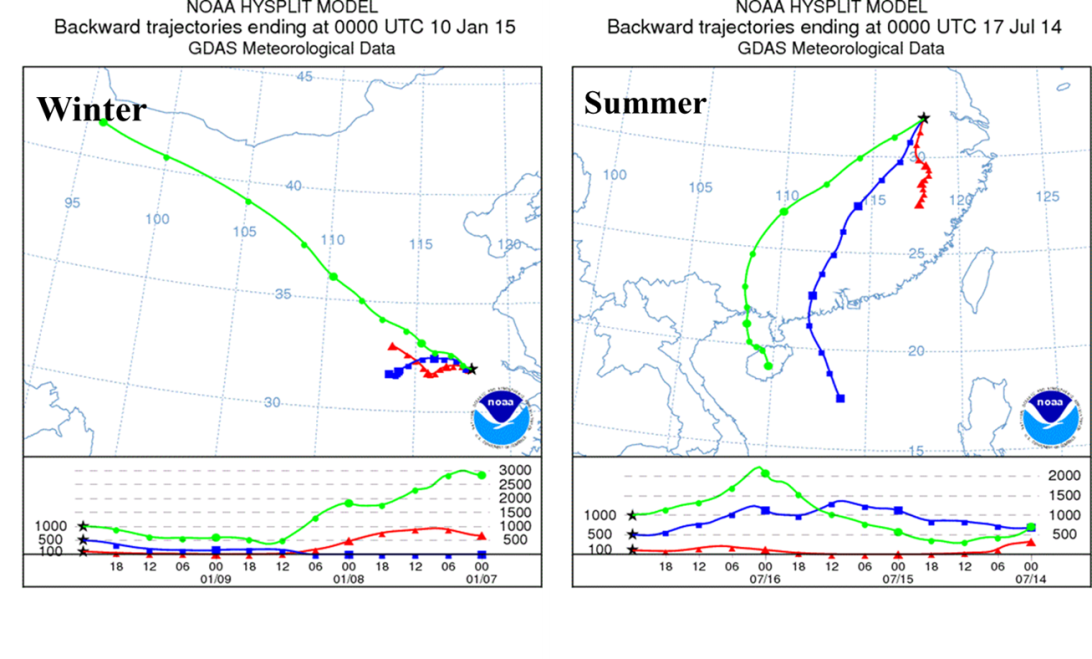


**Supplementary Figure S2.** 72-h backward air trajectories for the sampling site in Nanjing at 100, 500, and 1000 m above ground during the summer (15–17 July 2014) and winter (14–16 January 2015) sampling periods. The maps were generated by the authors using the HYSPLIT model on the READY web site (<http://ready.arl.noaa.gov/HYSPLIT.php>).

**Supplementary Figure S3. Location of the sample site**. Figure created by the authors using ArcGIS 10.2.2 (www.esri.com) and Microsoft Office 2013(<https://products.office.com/zh-cn/buy/office>).

**Supplementary Table S1**

Pb isotope ratios in the summer and winter PM_2.5_ samples collected in Nanjing. Daytime (dt) samples were collected between 08:00 and 18:00, and nighttime (nt) samples between 19:00 and 07:00.

|  | ^207^Pb/^206^Pb | ^208^Pb/^206^Pb |
| --- | --- | --- |
| **Summer** | | |
| **Daytime** |  |  |
| Mean±S.D. | 0.8554±0.0031 | 2.1012±0.0089 |
| Min | 0.8497 | 2.0834 |
| Max | 0.8589 | 2.1119 |
| **Nighttime** |  |  |
| Mean±S.D. | 0.8521±0.0032 | 2.0828±0.0102 |
| Min | 0.8499 | 2.0828 |
| Max | 0.8595 | 2.1194 |
| **All summer samples** |  |  |
| Mean±S.D. | 0.8551±0.0031 | 2.0968±0.0094 |
| Min | 0.8497 | 2.0828 |
| Max | 0.8595 | 2.1194 |
| **Winter** | | |
| **Daytime** |  |  |
| Mean±S.D. | 0.8545±0.0026 | 2.0923±0.0103 |
| Min | 0.8492 | 2.0718 |
| Max | 0.8584 | 2.1070 |
| **Nighttime** |  |  |
| Mean±S.D. | 0.8558±0.0032 | 2.0922±0.00940 |
| Min | 0.8516 | 2.0785 |
| Max | 0.8621 | 2.1049 |
| **All winter samples** |  |  |
| Mean±S.D. | 0.8550±0.0029 | 2.0923±0.0097 |
| Min | 0.8492 | 2.0718 |
| Max | 0.8621 | 2.1070 |
| **All PM_2.5_ samples** |  |  |
| Mean±S.D. | 0.8551±0.0029 | 2.0945±0.0097 |
| Min | 0.8492 | 2.0718 |
| Max | 0.8621 | 2.1194 |

**Supplementary Table S2**

**Correlation coefficients (R) for the relationships between the PM_2.5_ concentrations, the trace metal concentrations, and the magnetic parameters.**

|  | PM_2.5_ | As | Cd | Co | Cr | | Cu | | Mn | | Ni | | Pb | | Sr | | Ti | | V | | Zn | | Fe | | χ_LF_ | | SIRM | | HIRM | | χ_ARM_ | | χ_ARM_/χ_LF_ | | χ_ARM_/SIRM | | SIRM/χ_LF_ | | S-ratio | L-ratio |
| --- | --- | --- | --- | --- | --- | --- | --- | --- | --- | --- | --- | --- | --- | --- | --- | --- | --- | --- | --- | --- | --- | --- | --- | --- | --- | --- | --- | --- | --- | --- | --- | --- | --- | --- | --- | --- | --- | --- | --- | --- |
| PM_2.5_ | 1 |  |  |  |  | |  | |  | |  | |  | |  | |  | |  | |  | |  | |  | |  | |  | |  | |  | |  | |  | |  |  |
| As | -0.339 | 1 |  |  |  | |  | |  | |  | |  | |  | |  | |  | |  | |  | |  | |  | |  | |  | |  | |  | |  | |  |  |
| Cd | -0.089 | 0.194 | 1 |  |  | |  | |  | |  | |  | |  | |  | |  | |  | |  | |  | |  | |  | |  | |  | |  | |  | |  |  |
| Co | -0.294 | 0.346* | -0.049 | 1 |  | |  | |  | |  | |  | |  | |  | |  | |  | |  | |  | |  | |  | |  | |  | |  | |  | |  |  |
| Cr | -0.634** | 0.675** | 0.117 | 0.508** | 1 | |  | |  | |  | |  | |  | |  | |  | |  | |  | |  | |  | |  | |  | |  | |  | |  | |  |  |
| Cu | -0.417** | 0.701** | 0.155 | 0.387* | 0.794** | | 1 | |  | |  | |  | |  | |  | |  | |  | |  | |  | |  | |  | |  | |  | |  | |  | |  |  |
| Mn | -0.204 | 0.475** | 0.156 | 0.273 | 0.531** | | 0.649** | | 1 | |  | |  | |  | |  | |  | |  | |  | |  | |  | |  | |  | |  | |  | |  | |  |  |
| Ni | 0.231 | 0.528** | -0.091 | 0.290 | 0.251 | | 0.486** | | 0.404** | | 1 | |  | |  | |  | |  | |  | |  | |  | |  | |  | |  | |  | |  | |  | |  |  |
| Pb | -0.200 | 0.707** | 0.561** | 0.135 | 0.488** | | 0.608** | | 0.523** | | 0.212 | | 1 | |  | |  | |  | |  | |  | |  | |  | |  | |  | |  | |  | |  | |  |  |
| Sr | -0.478** | 0.374* | 0.191 | 0.628** | 0.534** | | 0.446** | | 0.220 | | -0.012 | | 0.230 | | 1 | |  | |  | |  | |  | |  | |  | |  | |  | |  | |  | |  | |  |  |
| Ti | -0.423** | 0.513** | 0.018 | 0.407** | 0.732** | | 0.754** | | 0.599** | | 0.364* | | 0.299 | | 0.426** | | 1 | |  | |  | |  | |  | |  | |  | |  | |  | |  | |  | |  |  |
| V | -0.517** | 0.251 | 0.133 | 0.683** | 0.583** | | 0.474** | | 0.387* | | -0.011 | | 0.240 | | 0.742** | | 0.470** | | 1 | |  | |  | |  | |  | |  | |  | |  | |  | |  | |  |  |
| Zn | -0.386* | 0.555** | 0.426** | 0.23 | 0.660** | | 0.707** | | 0.474** | | 0.167 | | 0.665** | | 0.517** | | 0.458** | | 0.549** | | 1 | |  | |  | |  | |  | |  | |  | |  | |  | |  |  |
| Fe(%) | -0.116 | 0.455** | 0.515** | 0.259 | 0.393* | | 0.436** | | 0.487** | | 0.192 | | 0.549** | | 0.327* | | 0.426** | | 0.381* | | 0.529** | | 1 | |  | |  | |  | |  | |  | |  | |  | |  |  |
| χ_LF_ | 0.089 | 0.042 | 0.568** | -0.09 | -0.049 | | 0.027 | | 0.364* | | -0.162 | | 0.387* | | 0.018 | | 0.054 | | 0.077 | | 0.274 | | 0.717** | | 1 | |  | |  | |  | |  | |  | |  | |  |  |
| SIRM | 0.023 | 0.065 | 0.557** | -0.089 | 0.009 | | 0.060 | | 0.348* | | -0.232 | | 0.422** | | 0.067 | | 0.121 | | 0.093 | | 0.321* | | 0.736** | | 0.968** | | 1 | |  | |  | |  | |  | |  | |  |  |
| HIRM | 0.155 | 0.076 | 0.573** | -0.130 | -0.075 | | 0.020 | | 0.298 | | -0.136 | | 0.409** | | -0.040 | | 0.096 | | -0.016 | | 0.227 | | 0.696** | | 0.913** | | 0.952** | | 1 | |  | |  | |  | |  | |  |  |
| χ_ARM_ | 0.181 | -0.038 | 0.533** | -0.115 | -0.149 | | -0.064 | | 0.279 | | -0.149 | | 0.291 | | -0.050 | | -0.036 | | 0.039 | | 0.182 | | 0.672** | | 0.979** | | 0.932** | | 0.894** | | 1 | |  | |  | |  | |  |  |
| χ_ARM_/χ_LF_ | 0.339* | -0.207 | -0.205 | 0.049 | -0.308 | | -0.244 | | -0.266 | | 0.165 | | -0.296 | | -0.121 | | -0.297 | | -0.040 | | -0.295 | | -0.194 | | -0.199 | | -0.230 | | -0.150 | | -0.016 | | 1 | |  | |  | |  |  |
| χ_ARM_/SIRM | 0.277 | -0.074 | -0.190 | 0.083 | -0.189 | | -0.128 | | 0.118 | | 0.389* | | -0.252 | | -0.194 | | -0.267 | | -0.075 | | -0.252 | | -0.195 | | -0.114 | | -0.290 | | -0.236 | | 0.012 | | 0.530** | | 1 | |  | |  |  |
| SIRM/χ_LF_ | -0.112 | -0.024 | 0.017 | -0.086 | 0.086 | | 0.022 | | -0.266 | | -0.316* | | 0.091 | | 0.119 | | 0.198 | | 0.011 | | 0.088 | | 0.035 | | -0.094 | | 0.115 | | 0.112 | | -0.118 | | -0.014 | | -0.798** | | 1 | |  |  |
| S-ratio | -0.414** | 0.082 | -0.251 | 0.284 | 0.252 | | 0.156 | | 0.077 | | 0.014 | | -0.061 | | 0.247 | | -0.025 | | 0.214 | | 0.134 | | -0.339* | | -0.386* | | -0.441** | | -0.633** | | -0.432** | | -0.164 | | 0.189 | | -0.276 | | 1 |  |
| L-ratio | 0.315* | -0.085 | 0.171 | -0.207 | -0.123 | | -0.190 | | -0.090 | | -0.042 | | 0.015 | | -0.187 | | -0.009 | | -0.067 | | -0.125 | | 0.29 | | 0.276 | | 0.303 | | 0.464** | | 0.332* | | 0.247 | | -0.079 | | 0.198 | | -0.903** | 1 |
| **. Correlation is significant at the 0.01 level (2-tailed). | | | | | |  | |  | |  | |  | |  | |  | |  | |  | |  | |  | |  | |  | |  | |  | |  | |  | |  |  | |
| *. Correlation is significant at the 0.05 level (2-tailed). | | | | | |  | |  | |  | |  | |  | |  | |  | |  | |  | |  | |  | |  | |  | |  | |  | |  | |  |  | |

Supplementary text

Statistical analysis. To differentiate between anthropogenic influences and natural background levels of trace metals in PM_2.5_, an enrichment factor (EF) was calculated. Al, Zr, Fe, Sc, and Ti are often used as reference elements for EF calculations^1^. In this study, Ti was assumed to be a conservative element and used for the normalization in the crust. The EF values were calculated using Eq. ( 1).

$EF=(\text{C}_{n}/\text{C}_{ref})_{sample}/(\text{B}_{n}/\text{B}_{ref})_{background}$ (1)

where (*C*_n_/*C*_ref_)_sample_ and (*B*_n_/*B*_ref_)_background_ are the concentration ratios between the target element (n) and the reference element (*ref* = Fe) in the PM_2.5_ samples and in the background topsoil, respectively. When EF *<*10, the element is minimally enriched and predominantly originated from natural sources (e.g., dust or soil material); when 10 < EF < 100, the element is moderately enriched; and when EF > 100, the element is greatly enriched and the trace metals were mostly contributed by anthropogenic sources (e.g., industries or vehicles)^2^.

In this study, the background trace metals concentrations in topsoil from Jiangsu Province (from the publication “Background values of soil elements in China”) were used^3^.

Multiple linear regression analysis was used to develop a linear model according to Eq. (2)

$Y=b_{0}+b_{1}X_{1}+b_{2}X_{2}+\cdots\text{+}b_{n}X_{n}$ (2)

where Y is the element concentration (i.e., the dependent variable), X_1_–X_n_ are the decision variables (the PM_2.5_ concentrations and the magnetic parameter values), b_0_–b_n_ are the coefficients of the decision variables, and b_0_ is the intercept of the equation. The decision variables were added to the models one by one through stepwise multiple linear regressions, ensuring that the regression equation contained only significant variables.

**Supplementary References**

1. Loska, K., Wiechuła, D., Pelczar, J. Application of enrichment factor to assessment of zinc enrichment/depletion in farming soils. *Commun. Soil Sci. Plan.* **36**, 1117–1128 (2005).
2. Wei F et al. Ambient concentrations and elemental compositions of PM_10_ and PM_2.5_ in four Chinese cities. *Environ. Sci. Technol.* **33**(23): 4188-4193 (1999)
3. CNEMC (China National Environmental Monitoring Center). Background values of soil elements in China 330–378 (Chinese Environmental Science Press, 1990).
